# Electrocardiographic Biomarkers for Detection of Drug-Induced Late Sodium Current Block

**DOI:** 10.1371/journal.pone.0163619

**Published:** 2016-12-30

**Authors:** Jose Vicente, Lars Johannesen, Meisam Hosseini, Jay W. Mason, Philip T. Sager, Esther Pueyo, David G. Strauss

**Affiliations:** 1 Division of Applied Regulatory Science, Office of Clinical Pharmacology, Office of Translational Sciences, Center for Drug Evaluation and Research, US Food and Drug Administration, Silver Spring, MD, United States of America; 2 Division of Biomedical Physics, Office of Science and Engineering Laboratories, Center for Devices and Radiological Health, US Food and Drug Administration, Silver Spring, MD, United States of America; 3 BSICoS Group, Aragón Institute for Engineering Research (I3A), IIS Aragón, University of Zaragoza, Zaragoza, Spain; 4 Cardiology Division, University of Utah, Salt Lake City, UT, United States of America; 5 Spaulding Clinical Research, West Bend, WI, United States of America; 6 Stanford University, Palo Alto, CA, United States of America; 7 Biomedical Research Networking Center in Bioengineering, Biomaterials and Nanomedicine (CIBER-BBN), Zaragoza, Spain; University at Buffalo - The State University of New York, UNITED STATES

## Abstract

**Background:**

Drugs that prolong the heart rate corrected QT interval (QTc) on the electrocardiogram (ECG) by blocking the hERG potassium channel and also block inward currents (late sodium or L-type calcium) are not associated with torsade de pointes (e.g. ranolazine and verapamil). Thus, identifying ECG signs of late sodium current block could aid in the determination of proarrhythmic risk for new drugs. A new cardiac safety paradigm for drug development (the “CiPA” initiative) will involve the preclinical assessment of multiple human cardiac ion channels and ECG biomarkers are needed to determine if there are unexpected ion channel effects in humans.

**Methods and Results:**

In this study we assess the ability of eight ECG morphology biomarkers to detect late sodium current block in the presence of QTc prolongation by analyzing a clinical trial where a selective hERG potassium channel blocker (dofetilide) was administered alone and then in combination with two late sodium current blockers (lidocaine and mexiletine). We demonstrate that late sodium current block has the greatest effect on the heart-rate corrected J-T_peak_ interval (J-T_peak_c), followed by QTc and then T-wave flatness. Furthermore, J-T_peak_c is the only biomarker that improves detection of the presence of late sodium current block compared to using QTc alone (AUC: 0.83 vs. 0.72 respectively, *p*<0.001).

**Conclusions:**

Analysis of the J-T_peak_c interval can differentiate drug-induced multichannel block involving the late sodium current from selective hERG potassium channel block. Future methodologies assessing drug effects on cardiac ion channel currents on the ECG should use J-T_peak_c to detect the presence of late sodium current block.

**Trial Registration:**

NCT02308748 and NCT01873950

## Introduction

The current regulatory paradigm for assessing torsade de pointes (torsade) risk of new drugs focuses on whether the new drug or its metabolites block the hERG potassium channel [1] and prolong the heart rate corrected QT interval (QTc) on the electrocardiogram (ECG) in the thorough QT study [2], or an equivalent ECG analysis. This paradigm can be considered successful because no new marketed drugs have been removed from the market because of unexpected QTc prolongation or torsade risk since its implementation in 2005 [3]. On the other hand, this approach may have prevented potentially effective medicines from reaching the market, sometimes inappropriately [3]. This is because there are QTc prolonging drugs that block the hERG potassium channel, but have low torsade risk (e.g. ranolazine [4], amiodarone [5], verapamil [6]). In addition to hERG potassium channel block, these drugs block other inward currents (i.e. late sodium or L-type calcium), which can prevent the occurrence of early afterdepolarizations, the triggers for torsade [7, 8].

In order to address this, a consortium of multiple global drug regulators (U.S. Food and Drug Administration (FDA), European Medicines Agency, Health Canada, Japan Pharmaceutical and Medical Devices Agency), industry and academia coordinated by multiple public-private partnerships and professional societies (Health and Environmental Sciences Institute, Safety Pharmacology Society, Cardiac Safety Research Consortium) are developing a new paradigm, the Comprehensive *in vitro* Proarrhythmia Assay (CiPA), which contains four components [9]. First, all new drugs will be evaluated for the effects on multiple human cardiac ion channels and currents (e.g. hERG potassium, late sodium, L-type calcium) in overexpression cell lines and the results integrated together in an *in silico* computational model of the ventricular cardiomyocyte. Second, the *in silico* model will output a proarrhythmia score to divide drugs into very low risk, intermediate risk and high risk categories. Third, *in vitro* stem cell-derived cardiomyocyte assays will be used to confirm the electrophysiologic drug effects from the ion channel experiments. Fourth, exposure-response modeling will be applied to human electrocardiograms (ECGs) from early Phase 1 studies to determine if there are unexpected ion channel effects compared to preclinical ion channel data, which might occur due to a human specific metabolite or protein binding. Under this paradigm, it will be important to differentiate QTc prolonging drugs with balanced ion channel effects, such as ranolazine (i.e. hERG potassium channel and late sodium current block), that are not associated with torsade from selective hERG potassium channel blockers, such as dofetilide, that are associated with torsade.

Two FDA-sponsored clinical trials have shown that drug-induced shortening of the heart rate corrected J-T_peak_ interval (J-T_peak_c) is a sign of late sodium current block [10, 11]. In addition, an analysis of 12 T-wave biomarkers from the first FDA-sponsored clinical trial (FDA study 1: NCT01873950) that included dofetilide, quinidine, ranolazine and verapamil demonstrated that drug-induced changes in multiple T-wave morphology biomarkers are directly related to the amount of hERG potassium channel block [12]. However, it was not clear if inward current block affected T-wave morphology biomarkers.

In this study, we assess drug effects on T-wave morphology biomarkers in the second FDA-sponsored clinical trial (FDA study 2: NCT02308748) that included two late sodium current blocking drugs (mexiletine and lidocaine) administered alone and in combination with a selective hERG potassium channel block (dofetilide). In addition, we rank eight ECG biomarkers by their ability to detect late sodium current block and determine the optimal combination of biomarkers to differentiate selective hERG potassium channel block from multichannel block involving the late sodium current.

## Methods

This study was approved by the US Food and Drug Administration Research Involving Human Subjects Committee (RIHSC 13-011D and 14-022D) and the local institutional review board (Chesapeake IRB). All subjects gave written informed consent and the study was performed at a Phase 1 clinic (Spaulding Clinical Research, West Bend, WI, USA).

### Clinical trial design

The study design of this Phase 1 clinical trial (FDA study 2: NCT02308748) has been described previously [11]. Briefly, this was a five-period, randomized, cross-over trial designed to study the ability of late sodium or calcium current block to balance the ECG effects of selective hERG potassium channel block. There was one week between treatment periods. In each treatment period subjects were dosed three times during the day with placebo, a selective hERG potassium channel blocker (dofetilide [Tikosyn, Pfizer, USA] or moxifloxacin) or a late sodium current blocker (mexiletine [Teva Pharmaceuticals USA Inc.,USA] or lidocaine [B. Braun Medical, Inc., USA]) alone or in combination (mexiletine combined with dofetilide, lidocaine combined with dofetilide), or a selective hERG potassium channel blocker (moxifloxacin) alone or in combination with a calcium channel blocker (diltiazem). Plasma drug concentration was measured using a validated liquid chromatography with tandem mass spectroscopy method by Frontage Laboratories (Exton, Philadelphia, PA). The present study focuses on the effects of late sodium current block when combined with selective hERG potassium channel block on eight ECG biomarkers. Therefore, time-points from the combination of diltiazem with moxifloxacin were not included in this study. One female was excluded from this analysis because in the evening time-point of the dofetilide alone arm, plasma dofetilide concentration was lower despite the evening oral dose, but she still had larger placebo- and baseline-corrected changes in all ECG biomarkers.

### ECG measurement methodology

Continuous ECG recordings were performed using the Mortara Surveyor system (Mortara, Milwaukee, WI, USA) sampled at 500 Hz with an amplitude resolution of 2.5 μV. Three 10 second non-overlapping 12-lead ECGs were extracted prior to the draw of each pharmacokinetic sample, based on heart rate stability and signal quality using Antares software (AMPS LLC, New York, NY, USA). All extracted 10s ECGs were up-sampled to 1000 Hz.

T-wave morphology biomarkers were automatically assessed in the 10s ECGs as previously described [12]. Briefly, T-wave flatness and asymmetry were automatically assessed with QTGuard+ (GE Healthcare, Milwaukee, WI, USA). T-wave amplitude as well as 30% of early and late repolarization duration (ERD_30%_ and LRD_30%_) [13] were automatically assessed with ECGlib [14]. The semi-automatic evaluation of QT, J-T_peak_ and T_peak_-T_end_ subintervals included in this analysis has been described elsewhere [10, 11].

### Analysis and correction of heart rate dependency

T-wave morphology biomarkers dependent on heart rate were corrected for heart rate as previously described [12]. Briefly, a population-based heart rate correction factor was developed using baseline data from the present study. In all subsequent analyses, the heart rate dependent biomarkers were corrected for heart rate using an exponential model (biomarker_c_ = biomarker/RR^α^), where the values of the α coefficient were 0.50 for T-wave flatness, 0.96 for the maximum magnitude of the T vector and 1.17 for the ventricular gradient. The heart rate correction was performed using PROC MIXED in SAS 9.3 (SAS Institute, Cary, NC), wherein a significance level of 0.05 was used to determine if there was a difference by sex.

### Statistical methods

We assessed whether there was a mitigation of dofetilide-induced ECG changes by either mexiletine or lidocaine at the population’s maximum plasma drug concentration (Cmax) time-point during the evening dose (Tmax). Mitigation was assessed using a paired t-test between the individual placebo-corrected changes from baseline with the combination vs. the individual predicted placebo-corrected changes from baseline at the individual measured dofetilide concentrations at Tmax in R 3.2.2 (R Foundation for Statistical Computing, Vienna, Austria). We assessed the magnitude of the mitigation for each ECG biomarker using Cohen’s d [15] effect size, which we also labeled as "negligible" (|d|<0.2), "small" (|d|<0.5), "medium" (|d|<0.8) or "large" (|d|≥0.8) [16] in R.3.2.2. The placebo-corrected change from baseline was computed using PROC MIXED in SAS 9.3, where the change from baseline for each ECG biomarker by time-point was the dependent variable and sequence, period, time, drug, and an interaction between treatment and time were included as fixed effects, and subject was included as a random effect. Afterwards, a linear-mixed effects model was used to evaluate the relationship between each of the ECG biomarkers and plasma concentrations for dofetilide and moxifloxacin alone. This was done using PROC MIXED in SAS 9.3 using a random effect on both intercept and slope (i.e., allowing each subject to have his or her own drug concentration-biomarker relationship). P values <0.05 were considered statistically significant without adjustment for multiplicity, and should be interpreted with an appropriate level of caution.

#### ECG signature of selective hERG potassium channel block

We used a previously described method [12] to assess the ECG signatures of selective hERG potassium channel block associated with dofetilide and moxifloxacin. Briefly, we used the concentration-response models to predict the individual drug-induced changes for QTc and each ECG biomarker at 25% increments of the population’s Cmax of each drug. Then, predicted changes together with 95% confidence intervals were plotted for each ECG biomarker vs. QTc.

#### Receiver operating characteristic (ROC) analysis

We developed multiple logistic regression models using one or two ECG biomarkers to classify selective hERG potassium channel block (dofetilide and moxifloxacin alone arms) vs. multichannel block with late sodium current inhibition (mexiletine with dofetilide and lidocaine with dofetilide arms) using placebo-corrected changes from baseline in each ECG biomarker pooled from all time-points (afternoon and evening time-points for dofetilide alone, mexiletine with dofetilide, and lidocaine with dofetilide; morning and afternoon time-points for moxifloxacin alone). Only biomarkers that had significant mitigation of dofetilide-induced changes by either mexiletine or lidocaine were used in the models built with two ECG biomarkers. We computed the receiver operating characteristic (ROC) area under the curve (AUC) to assess the performance of each model and 95% confidence intervals (CI) of AUC were computed with 2000 stratified bootstrap replicates. We compared the performances of the models using Delong’s test [17]. This analysis was done in R 3.2.2.

#### Decision tree

Lastly, we developed a C4.5 decision tree [18] to assess the relationship between the ECG biomarkers and selective hERG potassium channel block vs. multichannel block. The decision tree was developed using 10-fold cross-validation on all data from selective hERG potassium channel block (dofetilide [afternoon and evening time-points] and moxifloxacin [morning and afternoon] alone) vs. multichannel block (afternoon and evening time-points of mexiletine with dofetilide and lidocaine with dofetilide) (training set). We assessed the performance of the decision tree in the training set, but also using all data from our previous clinical study (FDA study 1: NCT01873950, validation set) [10], where each drug was labeled according to its corresponding ion channel current block at the population’s Cmax achieved in the clinical trial [12]. More specifically, dofetilide and quinidine were labeled as predominant hERG potassium channel blockers and ranolazine and verapamil as multichannel blockers. The decision tree was computed using the J48 algorithm in WEKA data mining software [19] version 3.6. The “positive” class for all classification methods (ROC-AUC and decision tree) was “multichannel block”.

## Results

The study included 21 healthy subjects (8 female). No serious adverse events were observed [11]. Table 1 reports the baseline characteristics of the subjects included in this analysis.

**Table 1:** Baseline characteristics.

	All subjects
	(N = 21)
**Demographic**	
Age (years)	26 ± 5
Female	8 (38%)
Body mass index (kg/m^2^)	23.7 ± 2.3
Heart rate (beats per minute)	60.7 ± 6.3
**QT and subintervals**	
QTc (ms)	397.1 ± 14.0
JT_peak_c (ms)	228.2 ± 18.2
T_peak_-T_end_ (ms)	82.2 ± 6.4
**T-wave morphology**	
T-wave flatness (d.u.)	0.43 ± 0.05
T-wave asymmetry (d.u.)	0.20 ± 0.00
ERD_30%_ (ms)	50.1 ± 7.4
LRD_30%_ (ms)	31.1 ± 5.3
T-wave amplitude (μV)	569.0 ± 149.0

Continuous variables are represented as mean ± SD. QTc, Fridericia’s heart rate corrected QT interval; J-T_peak_c, heart rate corrected J-T_peak_ interval; EDR_30%_, 30% of early repolarization duration; LRD_30%_, 30% of late repolarization duration; d.u., dimensionless units.

### Selective hERG potassium channel block: dofetilide and moxifloxacin

**Fig 1:**
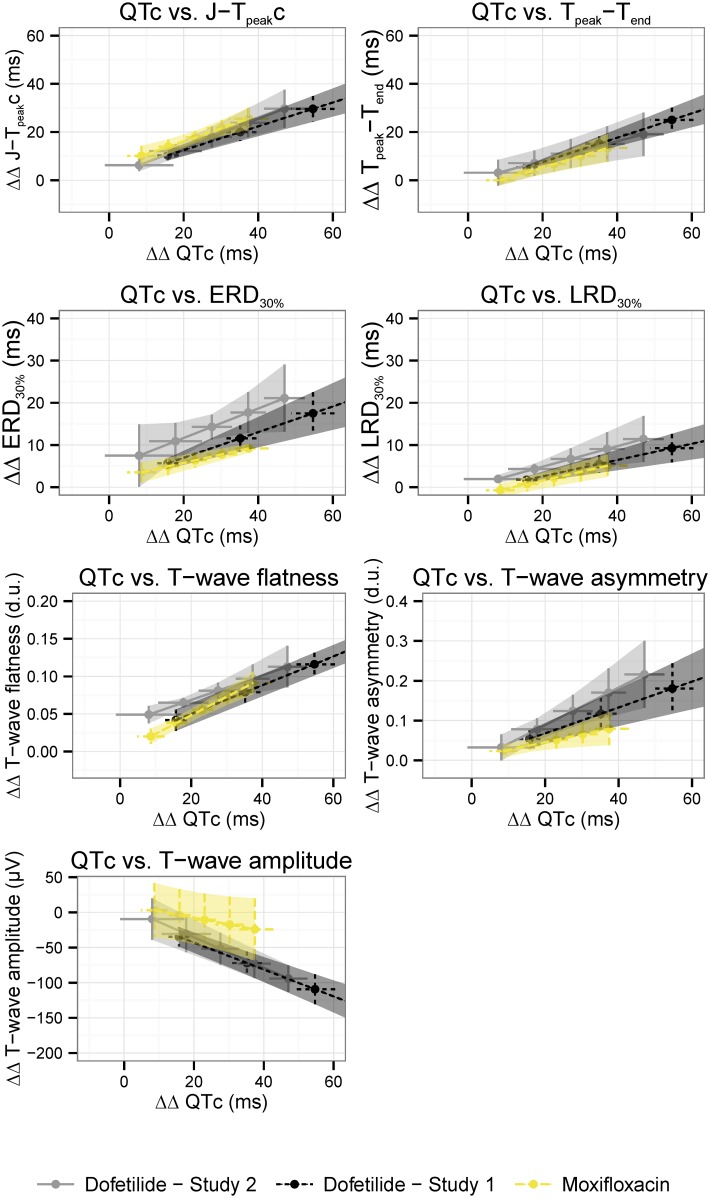
ECG “signature” of selective hERG potassium channel block. The ECG “signature” of selective hERG potassium channel block is shown as the relationship between predicted drug-induced placebo-corrected changes from baseline in QTc and (a) J-T_peak_c, (b) Tpeak-Tend, (c) 30% of early repolarization duration, (d) 30% of late repolarization duration, (e) T-wave flatness, (f) T-wave asymmetry and (g) T-wave amplitude. Average predictions (dots) and 95% confidence intervals (horizontal and vertical lines) are from the concentration-dependent models for QTc (x axis) and the different T-wave morphology biomarkers (y axis) at 25% increments of the population’s Cmax for dofetilide alone (light gray) and moxifloxacin (yellow) arms in this study together with the dofetilide arm from previous clinical study (Dofetilide—Study 1) [10, 12]. QTc, Fridericia’s heart rate corrected QT; J-T_peak_c, heart rate corrected J-T_peak_c interval.

At equivalent drug-induced QTc prolongation, T-wave morphology changes induced by moxifloxacin were similar to those caused by dofetilide. Moreover, the ECG “signature” of dofetilide was consistent with the ECG “signature” observed in our previous clinical study [12] (Fig 1).

### Mitigation of ECG changes by mexiletine and lidocaine

**Fig 2:**
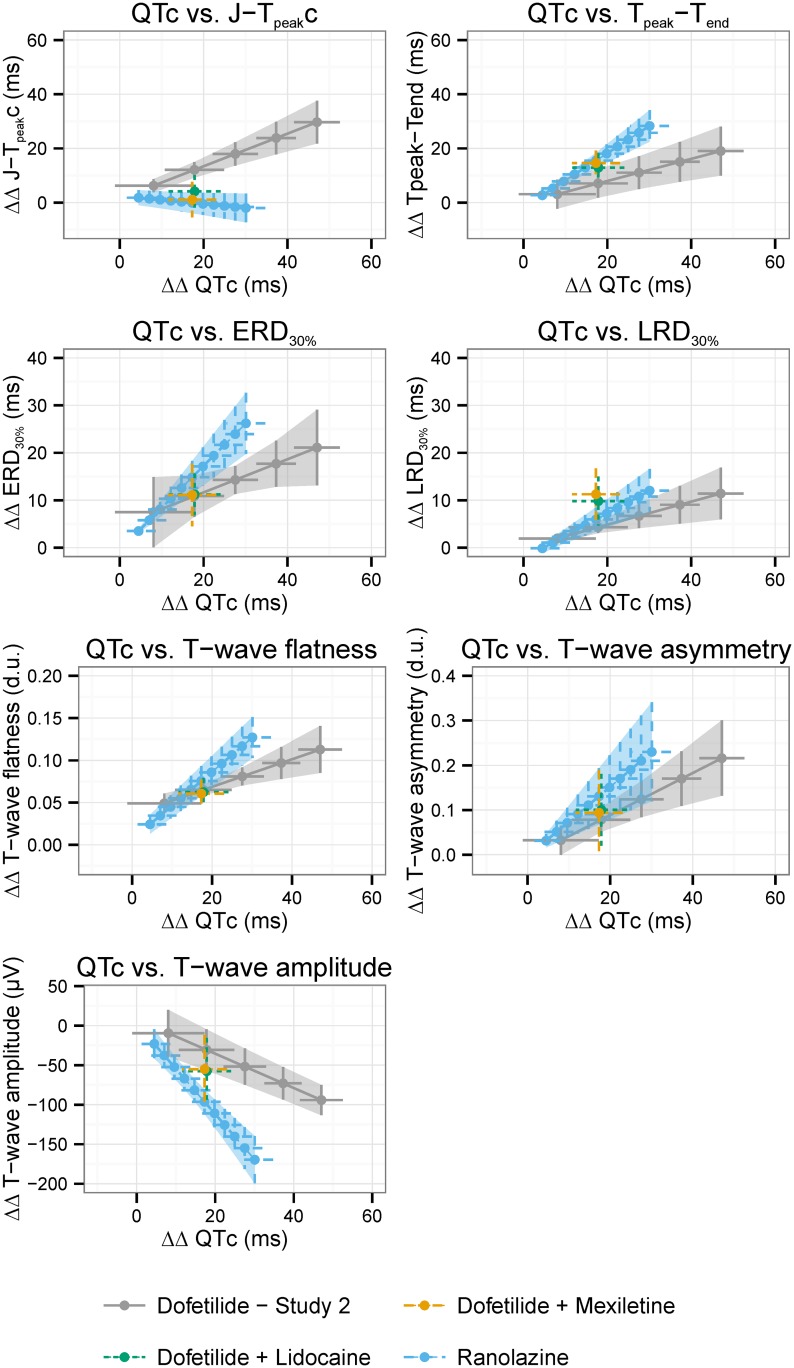
ECG “signatures” of dofetilide, ranolazine, mexiletine + dofetilide and lidocaine + dofetilide. The ECG “signatures” of dofetilide (light gray), ranolazine (blue), mexiletine + dofetilide (orange) and lidocaine + dofetilide (green) are shown as the relationship between predicted drug-induced placebo-corrected changes from baseline in QTc and (a) J-T_peak_c, (b) Tpeak-Tend, (c) 30% of early repolarization duration, (d) 30% of late repolarization duration, (e) T-wave flatness, (f) T-wave asymmetry and (g) T-wave amplitude. Average predictions (dots) and 95% confidence intervals (horizontal and vertical lines) are shown from the concentration-dependent models for QTc (x axis) and the different T-wave morphology biomarkers (y axis) at 25% increments of the population’s Cmax for dofetilide alone (light gray) arm in FDA study 2 and ranolazine (blue) arm from FDA study 1 [10, 12]. Average (dots) and 95% confidence intervals (vertical and horizontal bars) of placebo-corrected changes from baseline are shown for mexiletine + dofetilide (orange) and lidocaine + dofetilide (green). QTc, Fridericia’s heart rate corrected QT; J-T_peak_c, heart rate corrected J-T_peak_c interval.

When administered in combination with dofetilide, Cmax of mexiletine and lidocaine were achieved after the third dose of the corresponding treatment period (Table 2). Fig 2 shows the ECG signatures of dofetilide alone, mexiletine + dofetilide and lidocaine + dofetilide together with the ECG “signature” of ranolazine from our previous clinical study [12]. Late sodium current block by both mexiletine and lidocaine caused a numerical reduction of the ECG changes induced by dofetilide alone for all assessed ECG biomarkers except for 30% of late repolarization duration (Table 3). However, the changes were statistically significant only for QTc (*p*<0.001), J-T_peak_c (*p*<0.001) and T-wave flatness (*p*<0.001 and *p* = 0.015 for mexiletine + dofetilide and lidocaine + dofetilide, respectively).

**Table 2:** Cmax time-points (Tmax) and plasma drug concentrations of mexiletine and lidocaine when given in combination with dofetilide.

Arm	Tmax (hours)	Drug	Concentration (ng/mL)
Mexiletine + Dofetilide	13	Dofetilide	1.83 [1.71 to 1.95]
	13	Mexiletine	1426 [1312 to 1540]
Lidocaine + Dofetilide	12.5	Dofetilide	1.72 [1.52 to 1.91]
	12.5	Lidocaine	2261 [2072 to 2450]

Time: hours after first dose of day; Concentration: mean and 95% confidence intervals.

**Table 3:** Placebo- and baseline-corrected drug-induced ECG changes at Tmax.

Biomarker	Dofetilide	Combination	P
**Mexiletine + Dofetilide**			
QT and subintervals			
QTcF (ms)	45.2 [37.8 to 52.6]	17.3 [11.6 to 23]	**<0.001**
J-T_peak_c (ms)	29.1 [20.2 to 38]	1.1 [-5.5 to 7.7]	**<0.001**
T_peak_-T_end_ (ms)	17.9 [9.0 to 26.7]	14.6 [10.0 to 19.2]	0.36
T-wave morphology			
T-wave flatness (d.u.)	0.11 [0.08 to 0.14]	0.06 [0.04 to 0.08]	**<0.001**
T-wave asymmetry (d.u.)	0.20 [0.11 to 0.29]	0.09 [0.00 to 0.19]	0.056
ERD_30%_ (ms)	19.8 [11.5 to 28.1]	11.1 [4.5 to 17.7]	0.12
LRD_30%_ (ms)	9.8 [5.0 to 14.6]	11.3 [5.8 to 16.7]	0.57
T-wave amplitude (μV)	-87.9 [-106.3 to -69.5]	-55.1 [-98.3 to -11.9]	0.16
**Lidocaine + Dofetilide**			
QT and subintervals			
QTcF (ms)	45 [35.5 to 54.6]	17.9 [11.7 to 24.1]	**<0.001**
J-T_peak_c (ms)	28.3 [18.2 to 38.3]	4.1 [-1.9 to 10.2]	**<0.001**
T_peak_-T_end_ (ms)	18.4 [8.2 to 28.7]	12.9 [7.6 to 18.2]	0.23
T-wave morphology			
T-wave flatness (d.u.)	0.11 [0.07 to 0.14]	0.06 [0.05 to 0.08]	**0.015**
T-wave asymmetry (d.u.)	0.19 [0.09 to 0.29]	0.1 [0.02 to 0.18]	0.14
ERD_30%_ (ms)	18.4 [8.8 to 27.9]	11.2 [6.8 to 15.6]	0.19
LRD_30%_ (ms)	10.0 [4.5 to 15.4]	9.8 [4.6 to 15.0]	0.90
T-wave amplitude (μV)	-82.5 [-107.6 to -57.5]	-57.6 [-99.8 to -15.4]	0.27

Values reported as mean and 95% confidence intervals. QTc, Fridericia’s heart rate corrected QT interval; J-T_peak_c, heart rate corrected J-T_peak_ interval; T-wave flatness, heart rate corrected T-wave flatness; EDR30%, 30% of early repolarization duration; LRD30%, 30% of late repolarization duration; T-wave amplitude, heart rate corrected T-wave amplitude; d.u., dimensionless units.

### Ability of ECG biomarkers to detect late sodium current block

**Fig 3:**
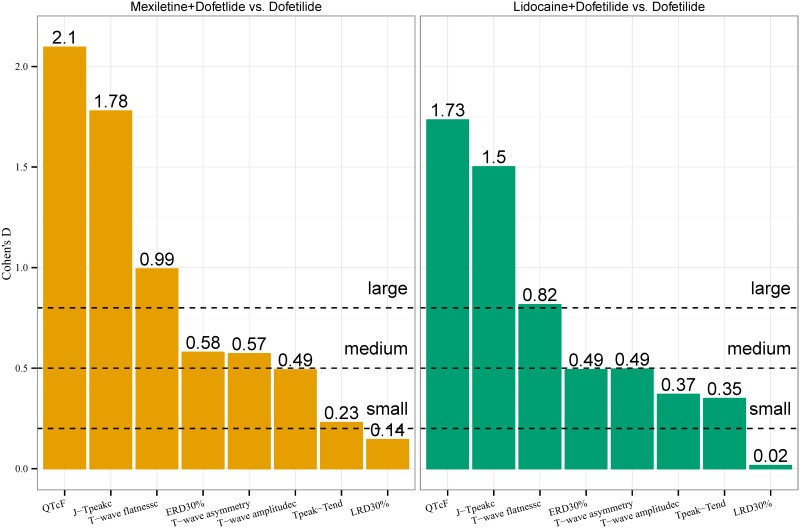
Cohen’s d effect size for each ECG biomarker. Cohen’s d effect size for each ECG biomarker for mexiletine + dofetilide and lidocaine + dofetilide vs. dofetilide alone.

Fig 3 shows Cohen’s d effect sizes for each ECG biomarker for mexiletine + dofetilide and lidocaine + dofetilide vs. dofetilide alone assessed at Tmax. While QTc, J-T_peak_c and T-wave flatness had “large” effect sizes, the effect sizes of QTc and J-T_peak_c were around two fold the effect size of T-wave flatness (Fig 3). All other five ECG biomarkers had “medium” or smaller effect sizes.

**Fig 4:**
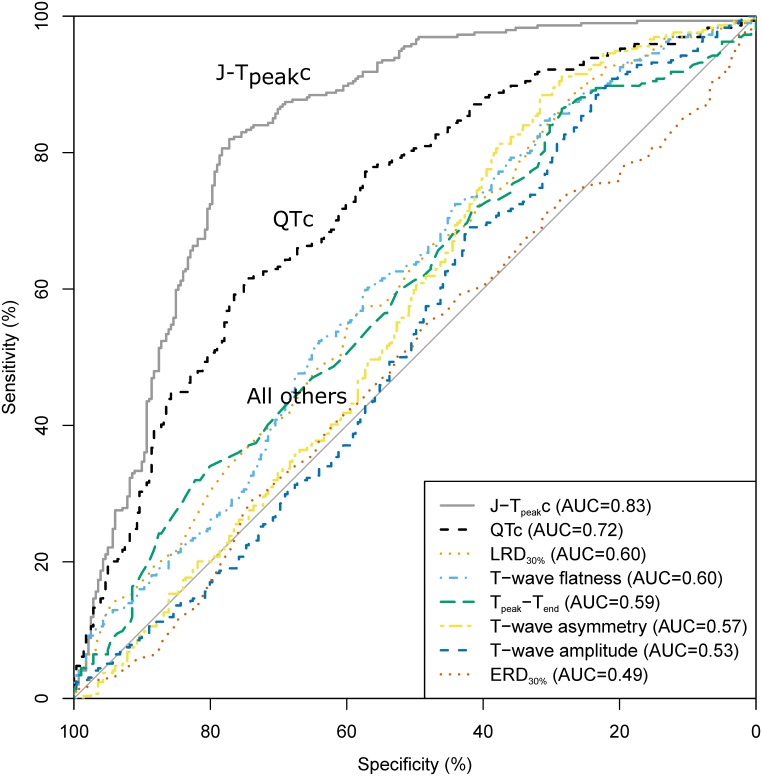
ROC curves for multiple logistic regression models. QTc, Fridericia’s heart rate corrected QT interval; J-T_peak_c, heart rate corrected J-T_peak_c interval; T-wave flatness, heart rate corrected T-wave flatness. Area under the curve of each model reported in parenthesis.

In the ROC-AUC analysis, J-T_peak_c had the largest AUC (0.83 [95% CI 0.80 to 0.87]) of all assessed ECG biomarkers (Fig 4). QTc had smaller AUC than J-T_peak_c (*p*<0.001) but greater than T-wave flatness (*p*<0.001). AUC of the model including both J-T_peak_c and QTc was not different than the AUC of J-T_peak_c alone (*p* = 0.63). Lastly, AUC of the model including both T-wave flatness and QTc was not different from QTc alone (*p* = 0.47). Table 4 summarizes AUC and the highest combined sensitivity + specificity from the ROC curve for all assessed models.

**Table 4:** Logistic regression by performance.

model	AUC	Sensitivity	Specificity
J-T_peak_c	0.83 [0.80 to 0.87]	0.82	0.77
QTc + J-T_peak_c	0.83 [0.80 to 0.87]	0.84	0.74
QTc + T-wave flatness	0.73 [0.69 to 0.77]	0.58	0.77
QTc	0.72 [0.68 to 0.77]	0.62	0.74
T-wave flatness	0.60 [0.56 to 0.65]	0.60	0.57
LRD_30%_	0.60 [0.56 to 0.65]	0.89	0.26
Tpeak-Tend	0.59 [0.55 to 0.64]	0.86	0.28
T-wave asymmetry	0.57 [0.52 to 0.62]	0.88	0.32
T-wave amplitude	0.53 [0.48 to 0.58]	0.89	0.23
ERD_30%_	0.49 [0.45 to 0.54]	0.54	0.49

Performance reported as area under the curve [AUC] to predict when additional inward current block is present. AUC brackets show 95% confidence intervals. Sensitivity and specificity are the highest combination of sensitivity + specificity on the ROC curve. QTc, Fridericia’s heart rate corrected QT interval; J-T_peak_c, heart rate corrected J-T_peak_ interval; EDR_30%_, 30% of early repolarization duration; LRD_30%_, 30% of late repolarization duration.

**Fig 5:**
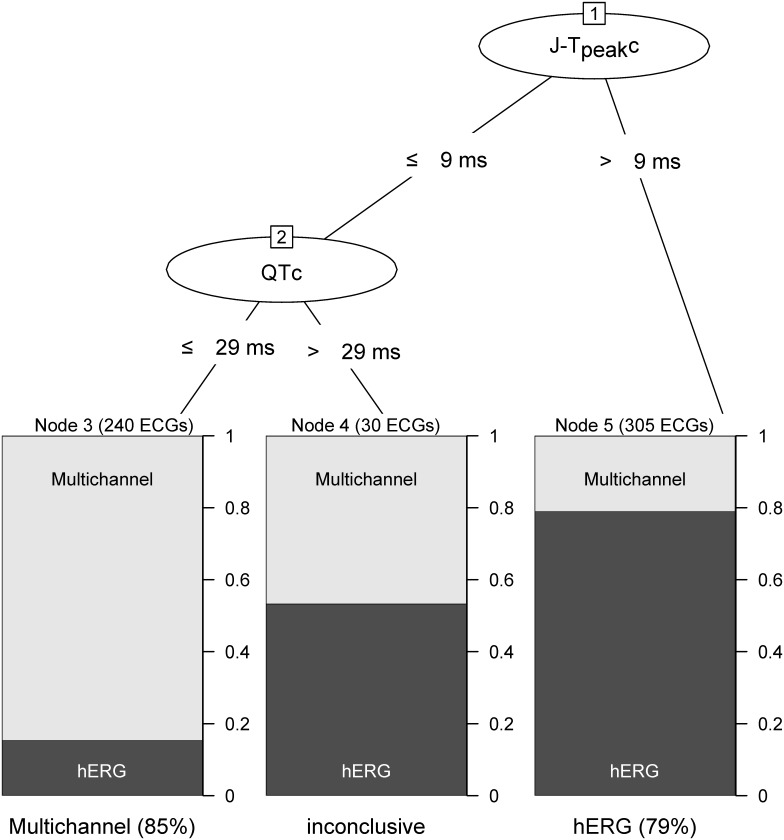
Decision tree. The decision tree classifies the drug effects on any ECG from any time point based on the time-matched placebo-corrected changes from baseline (ΔΔ) as selective of predominant hERG potassium channel block (right bin), multichannel block (left bin) or inconclusive (middle bin). More specifically, if ΔΔJ-T_peak_c is greater than 9ms the ECG is classified as predominant or selective hERG potassium channel block. If ΔΔ J-T_peak_c is less than or equal to 9ms and ΔΔQTc less than or equal to 29ms, then the ECG is classified as multichannel block. Otherwise the classification is inconclusive. The number of ECGs from each class in the training set is reported in parenthesis on top of each class bin. The percentage of ECGs correctly classified within each class is reported below each class bin. See text for more details on classification performance in both training (hERG [dofetilide and moxifloxacin] vs. multichannel [dofetilide + mexiletine and dofetilide + lidocaine]) and validation (hERG [dofetilide and quinidine] vs. multichannel [ranolazine and verapamil]) sets. QTc, Fridericia’s heart rate corrected QT interval; J-T_peak_c, heart rate corrected J-T_peak_ interval; hERG, selective or strong hERG potassium channel block; Multichannel, inward (late sodium or calcium) current and hERG potassium channel block.

Decision tree analysis selected the ECG biomarkers in the same order as the ROC-AUC analysis (J-T_peak_c, QTc and T-wave flatness). Fig 5 shows the decision tree developed using the best features in the ROC-AUC analysis (QTc and J-T_peak_c). The accuracy of the decision tree differentiating multichannel block from hERG potassium channel block was 0.80 [0.76 to 0.83] (sensitivity 0.85, specificity 0.77) in the training set and 0.83 [0.81 to 0.85] in the validation set (sensitivity 0.83, specificity 0.82).

## Discussion

In this study we assessed the effects of drug-induced late sodium current block on ECG changes caused by drug-induced selective hERG potassium channel block. The ECG signature of selective hERG potassium channel block was consistent between dofetilide and moxifloxacin and reproducible across studies for dofetilide. QTc, J-T_peak_c and T-wave flatness were the only three ECG biomarkers for which late sodium current block (mexiletine, lidocaine) caused statistically significant mitigation of changes induced by selective hERG potassium channel block (dofetilide). ROC-AUC analysis showed that J-T_peak_c was the best ECG biomarker for detecting late sodium current block, followed by QTc and then T-wave flatness. While the specific thresholds may not carry over to other studies, decision tree analysis showed that J-T_peak_c and QTc can be used to detect late sodium current block in the presence of QTc prolongation caused by selective hERG potassium channel block. Future methodologies assessing drug effects on the ECG, which could be applied in early Phase 1 studies under the CiPA initiative, should use J-T_peak_c to detect drug-induced late sodium current block.

The ECG signatures of selective hERG block were consistent between drugs (dofetilide and moxifloxacin) and reproducible for dofetilide across studies (Fig 1). Moxifloxacin is commonly used as a positive control in thorough QT studies [2] because it is a weak hERG potassium channel blocker at clinical concentrations, prolonging QTc by 10 to 14 ms (between 2 and 4 hours after 400mg oral dose), and has minimal torsade risk [20–22]. The similarity between moxifloxacin and dofetilide ECG “signatures” (Fig 1) suggests that the low torsade risk of moxifloxacin may be because moxifloxacin therapeutic doses cause limited hERG potassium channel block [11]. However, rare cases of moxifloxacin-induced torsade have been reported in patients at higher risk for torsade [23–26]. Therefore, in patients exposed to higher moxifloxacin concentrations with reduced “repolarization reserve” [27] or other comorbidities that increase risk of torsade [28], moxifloxacin may exhibit an ECG signature similar to dofetilide with larger QTc prolongation, larger T-wave morphology changes and higher risk for torsade.

There are multiple drugs that prolong QTc with minimal torsade risk [4–6], likely because they block inward currents [7, 29] in addition to the hERG potassium channel. We have previously shown that shortening of J-T_peak_c is a sign of inward (late sodium or L-type calcium) current block [10, 11, 30]. We have also shown that drug-induced changes in T-wave morphology biomarkers are directly related to the amount of hERG potassium channel block, and that multichannel blockers caused greater T-wave morphology changes than selective hERG potassium channel blockers at equivalent amount of QTc prolongation [12]. In this study we assessed which of all these ECG biomarkers can differentiate multichannel block (late sodium current block + hERG potassium channel block) from selective hERG potassium channel block on the ECG and therefore add value to QTc assessment. While there was a numerical reduction of hERG potassium channel induced changes for almost all ECG biomarkers after addition of late sodium current block, the effect was statistically significant only for QTc, J-T_peak_c and T-wave flatness (Table 3). This may have been due to larger inter-subject variability in the drug-induced changes in the T-wave morphology biomarkers compared to the more consistent response in QTc, J-T_peak_c and T-wave flatness (assessed as Cohen’s d effect size [15], Fig 5).

When comparing the ability of ECG biomarkers to detect the presence of late sodium current block, J-T_peak_c had the largest AUC in the ROC-AUC analysis. T-wave flatness did not add value to QTc assessment alone. Moreover, the J-T_peak_c model performed better than QTc alone or in combination with either J-T_peak_c or T-wave flatness (Table 4). The performance of the C4.5 decision tree developed with J-T_peak_c and QTc identifying inward current block using ECG data from FDA study 2 was similar to the performance achieved with ECG data of drugs from FDA study 1 [10] (accuracy of 0.80 [0.76 to 0.83] vs. 0.83 [0.81 to 0.84]). This suggests that, while appropriate thresholds still have to be established, a methodology using J-T_peak_c and QTc can identify balanced inward current (late sodium or calcium) and hERG potassium channel block vs. selective hERG potassium channel block, in particular in the presence of QTc prolongation.

The Comprehensive in Vitro Proarrhythmia Assay (CiPA) initiative [9] proposes an assessment of actual torsade risk beyond hERG potassium channel block and QTc prolongation. CiPA utilizes a mechanistically driven assessment of *in vitro* drug effects on multiple ion channels coupled with an *in silico* model of human cardiomyocytes and verification of predicted responses in human induced pluripotent stem cell derived cardiomyocytes. CiPA would include a clinical confirmation using Phase 1 ECGs to determine that no unanticipated clinical ECG changes are found compared to the preclinical ion channel data. Results of this study suggest that J-T_peak_c should be considered as a biomarker to detect late sodium current block in Phase 1 clinical studies under CiPA.

In addition to CiPA, there is another initiative that may affect the future of the current regulatory paradigm of proarrhythmic assessment of drugs. The Consortium for Innovation and Quality in Pharmaceutical Development and the Cardiac Safety Research Consortium (IQ-CSRC) recently demonstrated that exposure-response analysis of ECG and pharmacokinetic data from first-in-human studies can be used to assess drug-induced QTc prolongation with high confidence. The ICH E14 questions and answers (Q&A) were revised recently with harmonized guidance on how to use exposure-response modeling of QTc data (Q&A # 5.1) [31]. This ‘Early QT assessment’ could be used *in lieu* of thorough QT studies in some cases [32], which could potentially reduce drug development costs. However, this approach does not address the distinction between QTc prolonging drugs that selectively block the hERG potassium channel (high torsade risk) from QTc prolonging drugs that block inward currents in addition to the hERG potassium channel (low torsade risk) [9]. Thus, the ability of J-T_peak_c to detect inward current block in small sample sizes should be assessed and considered as a potential enhancement of the IQ-CSRC proposed paradigm.

Validation and implementation strategies for confirming CiPA preclinical findings using ECG data in small sample size Phase 1 clinical studies were discussed in a public workshop sponsored by the CSRC in April 2016. There is ongoing validation work on a large number of prior clinical studies with drugs that block individual and multiple ion channels. In addition, a statistical framework that can be applied in exposure-response analysis similar to the IQ-CSRC paradigm is being developed. Lastly, we are designing a confirmatory prospective clinical study with a small sample size design similar to first-in-human single or multiple ascending dose studies. It is anticipated that this prospective study will include a combination of selective hERG potassium channel blockers, multichannel blockers and no ion channel effect drugs [33].

In order to facilitate J-T_peak_c interval assessment in clinical drug trials, we have developed an automated algorithm for assessment of the J-T_peak_c interval. The automated algorithm can reproduce the semi-automated measurements of 8 drugs and 3 drug combinations from the two FDA-sponsored clinical trials. An implementation of this algorithm is being released as open-source code [34].

### Limitations

Classes in the training set were defined as ‘selective hERG potassium channel block’ vs. ‘late sodium current + hERG potassium channel block’, while in the validation set, the corresponding classes are predominant hERG block (dofetilide, quinidine) and multichannel block (ranolazine [late sodium current + hERG potassium channel block] and verapamil [strong calcium current block with some hERG potassium channel block]). This results in a division of the drugs in the test set into those with high torsade risk and predominant hERG block (dofetilide and quinidine) vs. low torsade risk and balanced inward and outward current block (ranolazine and verapamil). Thresholds of the decision tree should be interpreted with caution because differences in statistical methods may result in different values.

### Conclusion

J-T_peak_c was the best of the eight studied ECG biomarkers for detecting late sodium current block. ROC-AUC and decision tree analysis showed that an integrated assessment of J-T_peak_c and QTc can differentiate drug-induced multichannel block from selective hERG potassium channel block. Future methodologies assessing drug effects on cardiac ion channel currents on the ECG should consider J-T_peak_c to detect the presence of late sodium current block.
