# Correction: Electrocardiographic Biomarkers for Detection of Drug-Induced Late Sodium Current Block

**DOI:** 10.1371/journal.pone.0197952

**Published:** 2018-05-21

**Authors:** Jose Vicente, Lars Johannesen, Meisam Hosseini, Jay W. Mason, Philip T. Sager, Esther Pueyo, David G. Strauss

The funding information is incomplete. The complete funding information is as follows: This project was supported by FDA's Critical Path Initiative, Office of Women's Health and appointments to the Research Participation Programs at the Oak Ridge Institute for Science and Education through an interagency agreement between the Department of Energy and FDA. Pueyo is funded by Ministerio de Economía y Competitividad (MINECO), Spain, under project TIN2013-41998-R, by the European Research Council (ERC) through project ERC-2014-StG 638284 and by Grupo Consolidado BSICoS from DGA (Aragón) and European Social Fund (EU).
